# *ATAD3* duplications bridge mitochondrial diseases and Aicardi–Goutières syndrome

**DOI:** 10.1111/dmcn.16414

**Published:** 2025-07-15

**Authors:** Pauline Planté‐Bordeneuve, Claire‐Marine Bérat, Sylvain Hanein, Cyril Gitiaux, ATAD3 Study Group, Brian Sperelakis‐Beedham, Zahra Assouline, Marie‐Thérèse Abi‐Warde, Rukhshona Abdullazoda, Agnès Guichet, Céline Bris, Aurélien Caux, Marlène Rio, Jérémy Bertrand, Pauline Gaignard, Alice Lepelley, Arnold Munnich, Manuel Schiff, Isabelle Desguerre, Agnès Rötig, Nathalie Boddaert, Giulia Barcia, Julie Steffann, Isabelle Desguerre, Agnès Rötig, Nathalie Boddaert, Giulia Barcia

**Affiliations:** ^1^ Service de Médecine Génomique des Maladies Rares APHP Centre, Hôpital Necker‐Enfants Malades Paris France; ^2^ Reference Center for Mitochondrial Disorders Necker Hospital, APHP, Université Paris Cité Paris France; ^3^ Bioinformatic Platform Institute of Genetic Diseases, INSERM UMR1163, Institut Imagine, Université Paris Cité Paris France; ^4^ Paediatric Neurology Department Necker Hospital, APHP Centre, Université Paris Cité Paris France; ^5^ Université Paris Cité, Imagine Institute, INSERM UMR 1163 Paris France; ^6^ Paediatric Radiology Department APHP, Hôpital Necker‐Enfants Malades, Université Paris Cité, Institut Imagine, INSERM U1163 and U1299 Paris France

## Abstract

A recurrent 68‐kb heterozygous duplication of the *ATAD3* locus has been implicated in a mitochondrial disorder characterized by prenatal or neonatal onset and rapidly fatal course with cardiomyopathy, hyperlactataemia, cataract, and encephalopathy. We analysed the clinical, neuroimaging, and molecular spectrum associated with duplication of the *ATAD3* gene cluster in nine patients (four males, five females; age range: 3 days–3 years, median: 11 days, mean: 7.8 months, SD: 1 year 1 month). Five patients presented with prenatal signs (intrauterine growth restriction in four of nine and cardiac abnormalities in three of nine) leading to medical termination of pregnancy in one case. All live‐born children presented with neonatal hypotonia, frequently associated with cardiomyopathy (five of eight), cataract or corneal opacities (five of eight), and hyperlactataemia (six of eight). Two patients carrying distinct duplications exhibited a long survival (>2 years) and presented with major progressive brain atrophy with epileptic encephalopathy. We documented elevated cerebrospinal fluid neopterin in one and increased cerebrospinal fluid alpha‐interferon activity in the other. Brain magnetic resonance imaging showed white matter T2 hyperintensity (seven of seven) and temporal cystic leukoencephalopathy (five of seven). Nuclear magnetic resonance spectroscopy showed a lactate peak in five of five patients; brain computed tomography showed basal ganglia calcifications in two of three patients. In this study, we expand the clinical spectrum of *ATAD3* duplications, including prolonged survival and severe neurological involvement with neuroimaging similarities to Aicardi–Goutières syndrome and more broadly interferonopathy. We suggest a putative common mechanism that involves mitochondrial nucleic acid leakage and interferon response.

## Graphical abstract

ATAD3 locus duplications cause a severe neonatal mitochondrial disorder with neuroimaging features resembling interferonopathies, and suggest a mitochondrial nucleic acid‐triggered interferon response.

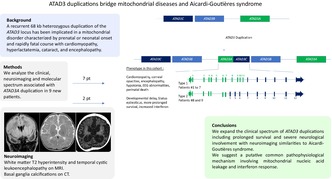

Plain language summary: https://onlinelibrary.wiley.com/doi/10.1111/dmcn.16446

**Abbreviations**
CSF: cerebrospinal fluid
IFN: interferon
PMD: primary mitochondrial disease

**What this paper adds**

- *ATAD3* duplication is linked to prolonged survival and severe neurological symptoms resembling Aicardi–Goutières syndrome.
- Mitochondrial nucleic acid leakage triggers an interferon‐mediated immune response.

With a prevalence of 1:2000 to 1:5000 live births, primary mitochondrial diseases (PMDs) caused by pathogenic variants in nuclear or mitochondrial genomes are among the most common inherited metabolic diseases.^1^ Over 400 disease‐causing nuclear and mitochondrial DNA genes are associated with PMDs.^2^ Among them, *ATAD3A* is localized in a locus containing three highly homologous tandemly arrayed genes, that is, *ATAD3C*, *ATAD3B*, and *ATAD3A*, on chromosome 1p36.3.

*ATAD3A* is ubiquitously expressed and encodes the ATPase family AAA domain‐containing protein 3A, which localizes between the inner and outer mitochondrial membranes. Although its precise molecular functions are not fully understood, *ATAD3A* has been implicated in hormone‐induced steroidogenesis, mitochondrial DNA organization and segregation, mitochondrial translation, adipogenesis, lipid metabolism, iron and haeme homeostasis, and maintenance of the mitochondrial network.^3^, ^4^

In 2016, monoallelic and biallelic pathogenic variants of *ATAD3A* were reported in patients with PMD and global neurodevelopmental delay, hypotonia, axonal neuropathy, cerebellar atrophy, cataract, and hypertrophic cardiomyopathy.^5^ More recently, a recurrent de novo heterozygous 68‐kb duplication of the *ATAD3* locus was identified in 22 patients who died during the first weeks of life.^6^, ^7^ These patients presented with persistent hyperlactataemia, hypertrophic cardiomyopathy, cataract or corneal opacities, hypotonia, and encephalopathy. These *ATAD3* duplications result in a chimeric *ATAD3* locus and exert their pathogenic effect through a dominant‐negative mechanism.^6^ In this article, we describe nine unreported cases of duplications in the *ATAD3* locus, including a previously unknown clinical phenotype, with later onset and prolonged survival in two patients. These two patients carry a new duplication of the *ATAD3* locus.

## METHOD

Table 1 presents age at onset of clinical signs and the clinical course of nine unreported patients (four males, five females, age range: 3 days–3 years, median: 11 days, mean: 7.8 months, SD: 1 year 1 month) born to unrelated parents in eight families with various ancestries. Among them, two patients presented their first clinical symptoms after the neonatal period and survived up to 2 years and 3 years respectively (patients 8 and 9, Table 1).

**TABLE 1:** Genotype and phenotype description of nine new patients carrying a heterozygous *ATAD3* duplication.

Characteristic	Patient no. 1	Patient no. 2	Patient no. 3	Patient no. 4	Patient no. 5	Patient no. 6	Patient no. 7	Patient no. 8	Patient no. 9	Gunning et al.^7^	Frazier et al.^6^
Age at onset of clinical signs	Prenatal	5 hours	Prenatal	Prenatal	Prenatal	12 hours	Prenatal	5 months	2 months	Prenatal onset; 3/5; 60%	Prenatal onset; 9/17; 53%
Clinical signs at onset	IUGR; neonatal distress; hypotonia; cardiomyopathy	Neonatal distress; hypotonia; cardiomyopathy	IUGR; neonatal distress; hypotonia; cardiomyopathy	IUGR; neonatal distress; hypotonia; cardiomyopathy	IUGR; hypotonia	Hypotonia; myoclonic jerks; hypertrophic cardiomyopathy	Dilated cardiomyopathy	Developmental delay; status epilepticus	Developmental delay; status epilepticus		
Age at death	13 days	9 days	4 days	3 days	8 days	14 days	NA (medical pregnancy termination)	22 months	3 years	Before 2 months 5/5; 100%; mean 24 days; 3 days to 6 weeks	Before 2 months 16/16; 100%; mean 24.6 days; fetal death (2 months)
Genetic investigation	Genome sequencing	Genome sequencing	Targeted NGS panel	Targeted NGS panel	Targeted NGS panel	Targeted NGS panel	Targeted NGS panel	Targeted NGS panel	Targeted NGS panel	Exome sequencing	Long read sequencing
Type of *ATAD3A*/*ATAD3C* duplication	Type 1	Type 1	Type 1	Type 1	Type 1	Type 1	Type 1	Type 2	Type 2	NC	NC
HGVS nomenclature	Seq[GRCh38] dup(1)(p36.33p36.33) NC_00001.11:g.1456401_1524800dup	Seq[GRCh38] dup(1)(p36.33p36.33) NC_00001.11:g.1456301_1525000dup	Seq[GRCh37] dup(1)(p36.33p36.33) chr1:g.(?_1392426)_(1459911_?)dup	Seq[GRCh37] dup(1)(p36.33p36.33) chr1:g.(?_1391535)_(1459400_?)dup	Seq[GRCh37] dup(1)(p36.33p36.33) chr1:g.(?_1392426)_(1459911_?)dup	Seq[GRCh37] dup(1)(p36.33p36.33) chr1:g.(?_1392426)_(1459911_?)dup	Seq[GRCh37] dup(1)(p36.33p36.33) chr1:g.(?_1392426)_(1459911_?)dup	Seq[GRCh37] dup(1)(p36.33p36.33) chr1:g.(?_1389623)_(1456061_?)dup	Seq[GRCh37] dup(1)(p36.33p36.33) chr1:g.(?_1389623)_(1456061_?)dup	NC_000001.11 (GRCh38):1456616_1524663dup (pt1‐3, 5) and (GRCh38):1456890_1524937dup (pt 5	NC_000001.10 (GRCh37):g.1392270_1460317dup, NC_000001.10:g.1391996_1460043dup, NC_000001.10:1392294_1460341dup, NC_000001.10:1392560_1460670dup, NC_000001.10:1395419_1462718dup, NC_000001.10:13915576_1455623dup.
Heritability	De novo	De novo	NP	NA	De novo	Not maternally inherited; father NP	Not maternally inherited; father NP	De novo	NP	De novo; 5/5; 100%	De novo; 17/17; 100%
Neurological examination	Global hypotonia	Axial hypotonia; distal hypertonia	Global hypotonia	Global hypotonia	Axial hypotonia; distal hypertonia	Global hypotonia	NA	Global hypotonia	Axial hypotonia; distal hypertonia	5/5; 100%	6/16; 38%
Seizures	No	Yes, H12 coil	No	Yes	Yes	Yes	NA	Yes	Yes	2/4; 50%	8/16; 50%
EEG abnormalities	Discontinuous activity	Discontinuous activity	Discontinuous activity	Discontinuous activity	NP	Discontinuous activity	NA	Suppression burst	Suppression burst	3/3; 100%	6/6; 100%
Abnormal white matter abnormalities on MRI	Yes	Yes	Yes	NP	Yes	Yes	NA	Yes	Yes	3/3; 100%	6/6; 100%
Basal ganglia calcifications on CT	NP	NP	NP	No	NP	NP	NA	Yes	Yes	NP	½; 50%
Lactate peak on spectroscopy	Yes	Yes	Yes	NP	Yes	NP	NA	NP	Yes	2/2; 100%	4/4; 100%
Hypertrophic/dilated cardiomyopathy	Yes	Yes	Yes	Yes	NP	Yes	Yes	No	No	5/5; 100%	16/17; 94%
Cataract or corneal opacity	Yes	Yes	Yes	Yes	No	No	NA	No	Yes	5/5; 100%	11/16; 69%
Lactic acidosis	Yes	Yes	Yes	Yes	Yes	Yes	NA	NP	NP	¾; 75%	16/16; 100%

Abbreviations: CT, computed tomography; EEG, electroencephalogram; IUGR, intrauterine growth restriction; MRI, magnetic resonance imaging; NA, not applicable; NGS, next‐generation sequencing; NP, not performed.

**FIGURE 1:**
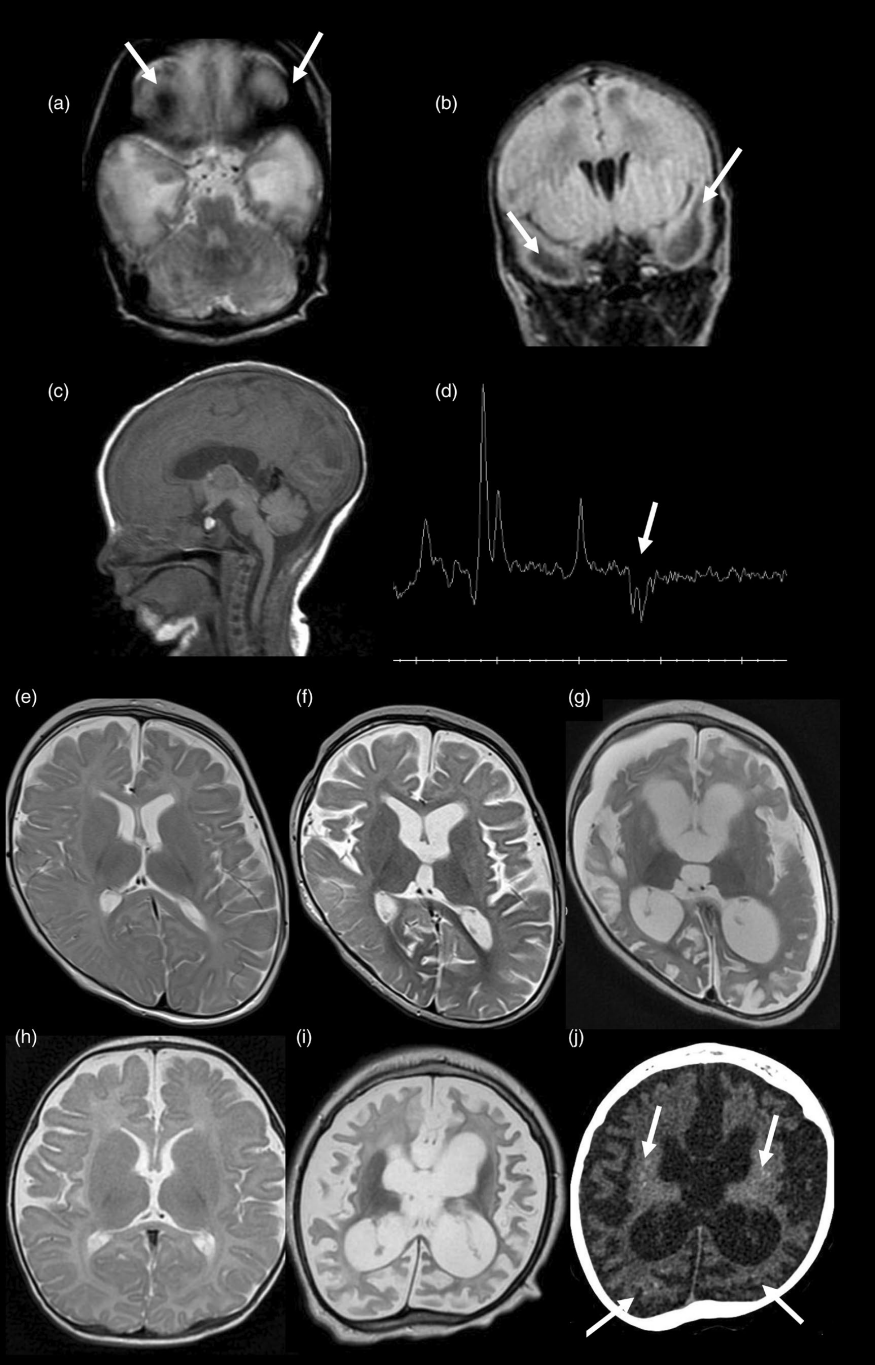
Neuroimaging features of patients carrying a heterozygous *ATAD3* duplication. (a) Axial T2‐weighted spectroscopy of patient 5 at 4 days of life. (b) Coronal fluid‐attenuated inversion recovery spectroscopy of patient 5 at 4 days of life. (c) Sagittal T1 spectroscopy of patient 5 at 4 days of life. (d) Magnetic resonance spectroscopy with an echo time of 144 ms of patient 5 at 4 days of life. (a,b) Magnetic resonance imaging (MRI) shows normal corpus callosum and cerebellum with cystic leukoencephalopathy in the bilateral temporal poles on T2 and fluid‐attenuated inversion recovery‐weighted sequences (a,b, arrows) with a lactate peak (d, arrow). (e) Axial T2‐weighted MRI of patient 8 at 5 months. (f) Axial T2‐weighted MRI of patient 8 at 9 months. (g) Axial T2‐weighted MRI of patient 8 at 11 months. The three MRIs show progressive atrophy with ventricular dilatation and enlargement of the pericerebral spaces. (h) Axial T2‐weighted MRI of patient 9 at 4 months of age. (i) Axial T2‐weighted MRIs of patient 9 at 2 years 11 months. (j) Computed tomography (CT) of patient 8. (h–i) MRI shows progressive atrophy with ventricular dilatation and enlargement of the pericerebral spaces. (j) CT shows bilateral basal ganglia and multiple cerebral calcifications (arrows).

Patient 8, a female, was born at term after uneventful pregnancy and delivery (Apgar score = 10/10). She was the first child of healthy parents with French ancestry. She presented with global hypotonia since birth. At age 5 months, she presented with left‐sided clonic status epilepticus. Subsequently, she presented with two episodes of right‐sided clonic status epilepticus at 8 months and 9 months. Seizures became daily from 10 months despite antiseizure medications (topiramate, phenytoin, clonazepam, carbamazepine), with associated psychomotor regression. At 10 months, she had no head control and presented with a tetrapyramidal syndrome. An electroencephalogram (EEG) at 1 year showed a suppression burst pattern. At 12 months, a ketogenic diet was introduced, which controlled the seizures, and she recovered eye contact. Cerebrospinal fluid (CSF) analysis showed normal glucose, protein, and cell counts. CSF neopterin concentration was elevated (354 nmol/L, normal range = 9–35 nmol/L) with normal neurotransmitter metabolites (5‐hydroxyindoleacetic acid, homovanillic acid, and 5‐methyltetrahydrofolate). Brain magnetic resonance imaging (MRI) revealed progressive cortico‐subcortical atrophy with white matter T2 and fluid‐attenuated inversion recovery hyperintensities and infratentorial atrophy (Figure 1e–g). Brain computed tomography (CT) was normal at 6 months but showed basal ganglia calcification at 10 months. The electrocardiogram and echocardiogram were normal. Mitochondrial respiratory chain analysis (liver and muscle) showed reduced activity of mitochondrial complexes II and III, along with a deficiency in complex I (not shown). Oxidative phosphorylation assembly analysed using Blue Native polyacrylamide gel electrophoresis on cultured fibroblasts was normal. Death occurred at 22 months.

Patient 9, a male born at 37 weeks to healthy, non‐consanguineous parents, presented at birth with axial hypotonia, peripheral hypertonia, and bilateral cataracts. From age 2 months, he developed clonic seizures, with several status epilepticus episodes during the first year. Neurological examination showed poor eye contact, absence of spontaneous movements, and a progressive tetrapyramidal syndrome. CSF analysis revealed elevated white blood cells (32–132/mm^3^ white cells, normal value <5/mm^3^ CSF) without evidence of infectious disease after extensive viral investigations. Type I interferon (IFN) activity was increased in the blood (12 IU/mL, normal value <2 IU/mL) and CSF (18–25 IU/mL, normal value <2 IU/mL). The EEG showed a burst‐suppression pattern. Brain MRI revealed progressive cerebral and infratentorial atrophy (Figure 1h,i). Brain CT showed periventricular and basal ganglia microcalcifications (Figure 1j). Nuclear magnetic resonance spectroscopy showed a lactate peak. Oxidative phosphorylation assembly in fibroblasts was normal. Based on the clinical and neuroimaging features, Aicardi–Goutières syndrome was suspected. Both electrocardiogram and echocardiogram were normal. The patient died aged 3 years. The clinical details of patients 1 to 7 are found in Appendix S1. The brain MRI and CT were reviewed by the same expert neuroradiologist (NB, Figure 1).

Genetic studies were performed using trio‐based targeted next‐generation sequencing in seven patients (patients 3–9) and using trio‐based genome sequencing in two patients (patients 1 and 2). Our targeted next‐generation sequencing panel targeted mitochondrial DNA and known nuclear PMD genes, including the three genes at the *ATAD3* locus (*ATAD3A*, *ATAD3B*, *ATAD3C*; Table S1). Genetic variants were classified according to the international guidelines of the American College of Medical Genetics and Genomics Laboratory Practice Committee Working Group and described according to the HGVS nomenclature guidelines (http://varnomen.hgvs.org/).

### Ethics statement

This study adhered to the Declaration of Helsinki (2013 revision) and was conducted in accordance with the French regulations. Our institution (AP‐HP) has a general privacy statement informing patients that their data may be used for scientific research (https://www.aphp.fr/protection‐des‐donnees‐personnelles‐information‐patient). All patients provided written informed consent for diagnosis and for the use of samples for research purposes. [Correction added on 3 November 2025 after first online publication: Ethics statement has been updated in this version.]

## RESULTS

Table 1 presents the clinical, neuroimaging, and genetic findings for nine unreported individuals carrying a duplication at the *ATAD3* locus. Among them, six of nine patients died in the perinatal period and two of nine patients died at 2 years and 3 years of age respectively. Some presented with fetal ultrasound evidence of intrauterine growth restriction (four of nine) and cardiac abnormalities (three of nine), leading to either premature labour induction or medical termination of pregnancy (patient 7). At live birth, eight of eight patients presented with neonatal hypotonia, which was associated with peripheral hypertonia in three of eight patients. Five patients (patients 1–4 and 6) exhibited hypertrophic or dilated cardiomyopathy, five of eight (patients 1–4 and 9) had bilateral cataracts or corneal opacities, six of eight (patients 1–6) had persistent hyperlactataemia, six of eight (patients 2, 4–6, and 8 and 9) had epileptic seizures, and all had an abnormal EEG, for example, discontinuous and poorly organized background activity.

Brain MRI was performed in seven patients and showed white matter T2 hyperintensity in all (Figure 1a–c), with temporal lobe cystic leukoencephalopathy in five of seven patients (Figure 1a). Nuclear magnetic resonance spectroscopy was performed in five patients and showed a lactate peak in all (Figure 1d). Brain CT was performed in three patients because of their haemodynamic instability; basal ganglia calcifications were found in two of three patients.

**FIGURE 2:**
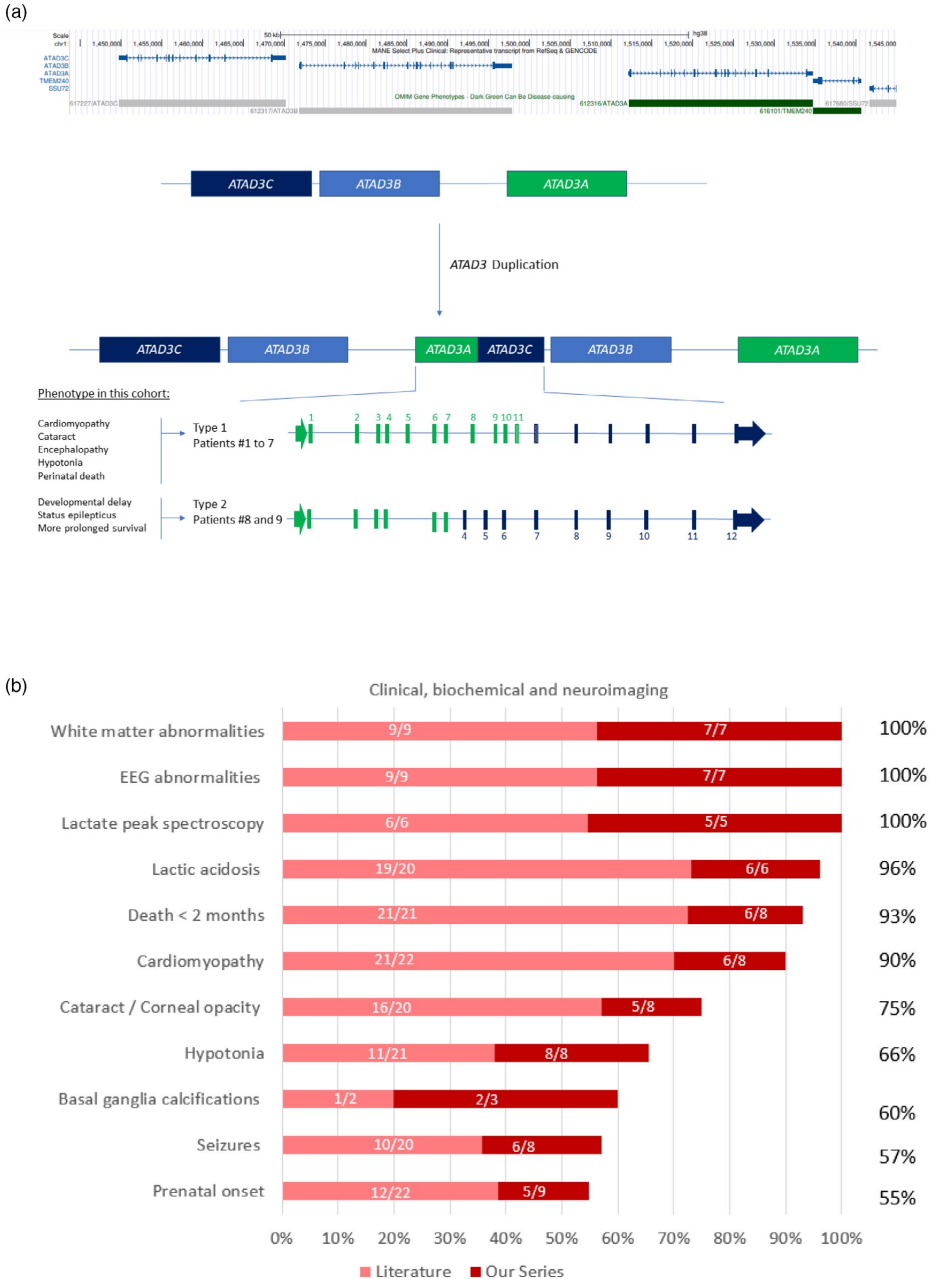
(a) *ATAD3* locus duplication. Schematic showing minimal localization of the *ATAD3A*/*ATAD3C* duplication. (b) Clinical summary of the literature and the patients in our case series with the *ATAD3* duplication. Abbreviation: EEG, electroencephalogram.

Genetic studies showed that seven of nine patients carried a duplication spanning exons 7 or 8 to 12 of *ATAD3C* to exons 1 to 10 or 11 of *ATAD3A*, including a complete duplication of *ATAD3B*, referred to as type 1 duplication (Figure 2a). Interestingly, patients 8 and 9 carried a different duplication, spanning from exons 4 to 12 of *ATAD3C* to exons 1 to 7 of *ATAD3A*, including a complete duplication of *ATAD3B*, referred to as type 2 duplication (Table 1 and Figure 2a). The precise breakpoints are not known due to our sequencing approach for patients 3 to 9 (targeted next‐generation sequencing). Studying parental DNA showed that the duplication occurred de novo in four of four. Segregation could not be studied in patient 3, 4, 6, 7, and 9. Finally, studying 10 flanking microsatellite markers in affected siblings 6 and 7 supported germline mosaicism in this family (Table 1).

According to the American College of Medical Genetics and Genomics criteria,^8^ these duplications are classified as pathogenic because they are absent from population databases (Genome Aggregation Database v4, Database of Genomic Variants) and occurred de novo when segregation analysis was performed. The duplication identified in patients 1 to 7 was already reported as pathogenic.^6^, ^7^ The duplication found in patients 8 and 9 is new but closely resembles those described in previous publications.^6^, ^7^

## DISCUSSION

Single‐nucleotide pathogenic *ATAD3* variants are known to cause an early and severe form of PMD. Heterozygous *ATAD3* duplications are responsible for a particularly recognizable presentation manifested by early‐onset cardiac involvement, with lactic acidosis, frequent cataract or corneal opacities, and frequent prenatal signs.^6^, ^7^ The clinical, biochemical, and neuroimaging data of the 31 patients are summarized in Figure 2b.

We report on nine new cases of heterozygous *ATAD3* duplication, including one case of germinal mosaicism in siblings.

We identified an *ATAD3* duplication spanning from exons 4 to 12 of *ATAD3C* to exons 1 to 7 of *ATAD3A* in two patients. Interestingly, and at odds with the hitherto reported *ATAD3* duplications, these two children presented with later disease onset, prolonged survival, and absent cardiac involvement. Their clinical course was dominated by severe neurological involvement and poor clinical outcome, with profound psychomotor delay, epileptic seizures, and recurrent episodes of status epilepticus, which was refractory to antiseizure medications. Neuroimaging showed a cystic leukoencephalopathy predominating in the temporal lobes, as well as progressive supratentorial and infratentorial atrophy. Nuclear magnetic resonance spectroscopy showed a lactate peak and brain CT identified basal ganglia calcifications in two‐thirds of patients.

Neuroimaging features, like those for patient 9, are observed in Aicardi–Goutières syndrome, congenital cytomegalovirus infection, and cystic leukoencephalopathy without megalencephaly due to *RNASET2* pathogenic variants (Figure 1).^9^

Aicardi–Goutières syndrome is a genetic disease resulting from altered nucleic acid processing, where self‐derived nucleic acid species induce a type I IFN‐mediated innate immune response, posited as pathogenic.^10^ Cytosolic nucleic acid released from mitochondria, and thus enhanced IFN signalling, can occur in mitochondrial diseases linked to mitochondrial DNA deletions (e.g. Pearson and Kearns–Sayre syndromes), which display clinical features overlapping with those seen in some type I interferonopathies, such as basal ganglia calcifications and skin lesions.^11^ Enhanced type I IFN signalling has also been observed in patients with *RNASET2* ^12^ and *PNPT1* leukoencephalopathy.^13^, ^14^ Pathogenic variants in *ATAD3A* reportedly trigger upregulated IFN‐stimulated gene expression and alpha‐IFN protein.^15^ Knockdown of *ATAD3A* in THP‐1 cells resulted in increased IFN signalling, which was mediated by cyclic GMP‐AMP synthase and stimulator of IFN genes.^15^ Duplications at the *ATAD3* locus, which are thought to act in a dominant‐negative manner,^6^ probably trigger a similar type I IFN‐mediated innate immune response. This is consistent with our findings of increased IFN activity in both CSF and blood in patient 9, as well as neuroimaging features reminiscent of disorders associated with enhanced IFN signalling in this cohort.

Recent studies suggested that pathogenic variants of the *ATAD3* locus may be among the top five causes of PMD.^6^, ^7^ However, these variants are probably underestimated because of the architectural complexity of the *ATAD3* locus. This cluster contains three highly homologous, tandemly duplicated genes, that is, *ATAD3A*, *ATAD3B*, and *ATAD3C*; it is difficult to characterize this cluster using routine molecular genetics technologies, such as chromosomal microarray analysis or targeted next‐generation sequencing and exome sequencing, which may not accurately characterize this region.^6^ In conclusion, our study broadens the clinical spectrum of *ATAD3* duplications and provides an insight into the pathophysiology of the disease and its link with Aicardi–Goutières syndrome and interferonopathies. It highlights the importance of searching for *ATAD3* locus duplications in cases of cerebral calcifications or elevated CSF IFN levels of unknown aetiology.

## CONFLICT OF INTEREST STATEMENT

None of the authors have any conflicts of interest to declare.

## Supporting information


**Table S1:** Next‐generation sequencing panel

**Appendix S1:** Sequencing methods

## Data Availability

The data that support the findings of this study are available from the corresponding author upon reasonable request.
