# Supplementary material for: ATAD3 duplications bridge mitochondrial diseases and Aicardi–Goutières syndrome
Source: Dev Med Child Neurol. 2025 Jul 15;68(2):287–94. doi: 10.1111/dmcn.16414 (PMC12766552; doi:10.1111/dmcn.16414)
Supplement: Supplementary file 2 — Appendix S1: Sequencing methods [file DMCN-68-287-s001.docx]

**Supplementary data**

**Sequencing methods**

For patients 1 and 2, DNA was sequenced by Genome Sequencing (GS). The trio GS was performed at the SeqOIA laboratory (https:// laboratoire-seqoia.fr/). The library was prepared using the NEB Next Ultra II End repair/A-tailing DNA Library Prep Kit (New England Biolab, Ipswich, MA, USA) and sequenced in paired ends (2 × 150 bp) using an Illumina Novaseq6000 platform. The reads were aligned to the reference human genome (GRCh38.92) using the BWA-MEM 0.7.15 software package. The GATK haplotype caller (v4.1.7.0; Broad Institute) was used to call the SNVs, and CNVnator (v0.4.1; Mark B, Yale University) was used for CNV calling. ClinSV^1^ a GS-based bio-informatic framework that combined different methods based on read depth, was used to annotate and prioritize structural variants, including CNVs. The resulting variants were then annotated with AnnotSVv2.5.1 in an in-house developed workflow (SeqOIA-IT platform).

For patients 3-9, DNA was sequenced using targeted next generation sequencing (TNGS) panel targeting mitochondrial DNA (mtDNA) and nuclear genes associated to PMD (supplementary Table 1), based on a sequence capture method (Agilent Technologies, Santa Clara, CA, USA). Paired-end sequence datasets from NextSeq Illumina runs were processed following three main steps: alignment against the human genome release hg19, variant calling of SNPs and small indels [using SAMtools^2^ GATK^3^], and variant annotation based on the Ensembl human database (GRCh37 release). Data were integrated in an in-house pipeline enabling copy number variant (CNV) analysis based on a double normalization of depth coverage.

We classified each variant according to the international guidelines of the American College of Medical Genetics and Genomics (ACMG) Laboratory Practice Committee Working Group and the ClinGen recommendations for the CNV Classification^4,5^

ANNOVAR was used to annotate variants according to the type of mutation, occurrence in a set of local control exomes, and GnomAD allele frequency. Potentially causal variants were defined according to the following set of stringency filters: a) present at < 1% minor allele frequency in GnomAD data set; b) present in fewer than 10 (alternatively) local control exomes (from unrelated projects, using similar sequencing chemistry and bioinformatics analysis pipeline); c) present either in annotated protein coding regions and nonsynonymous (missense, nonsense, frameshift) or in nearby adjacent intronic sequence.

**Clinical data**

Clinical data are resumed in Table 1.

Patient 1 (male) was the only child of healthy, non-consanguineous parents. The pregnancy was characterized by intrauterine growth restriction (IUGR). The patient was born at term with APGAR scores of 2/10/10, and exhibited global hypotonia and feeding difficulties. At birth, cardiac ultrasound revealed hypertrophic cardiomyopathy. He has a persistent hyperlactatemia during hospitalization. Ophthalmologic examination showed bilateral corneal opacity. Additional findings included hypospadias, hydrocele, and distinct facial features such as retrognathia. Brain MRI, performed at day 9, showed white matter abnormalities, bilateral pallidal hyperintensity on T2 sequences, and a lactate peak on spectroscopy. The EEG, performed at day 5, showed a disorganized background activity with lack of physiological rhythms.

mtDNA, exome sequencing and chromosomal microarray analysis were negative. Following the decision to limit active treatments due to the incurable nature of the condition and poor prognosis, the patient deceased at 13 days of life.

Patient 2 (female) was born at term after a regular pregnancy. Her non-consanguineous parents are healthy. She was born by dystocic delivery due to breech presentation. APGAR was 1/3/3/5 with suspected anoxic-ischemic encephalopathy and hyperlactatemia exceeding 20 mmol/L (normal range < 2.2 mmol/L) at 5 hours of life and persisting until her death. She was intubated and mechanically ventilated. She presented bilateral cataract, axial hypotonia and peripheral hypertonia. Her EEG showed a disorganized background activity without physiological rhythms and with bilateral temporal spikes. Clinically, she presented bilateral tonic contractions of the upper limbs at H12 of life.

Her brain MRI showed diffuse white matter abnormalities on T2 sequences, and spectroscopy revealed a significant lactate peak at 6 days of life. Severe biventricular hypertrophic cardiomyopathy was detected on echocardiography at 6 days. The child developed an oligo-anuric renal failure. Hemodynamic deterioration in a context of sepsis led to death at 9 days of life.

Urinary amino acid chromatography reveals massive hyperlactaturia, significant ketosis, and elevated levels of fumaric, malic, 3-methylglutaconic, phenyllactic, and phenylpyruvic acids. BN-PAGE on fibroblasts was normal. No accumulation of long-chain fatty acids was detected.

Patient 3 was a female born at 36+5 weeks of gestation via caesarean section, which was performed due to abnormal foetal cardiac rhythm. The pregnancy was complicated by IUGR. The patient is the third child of consanguineous parents, with two healthy siblings. The mother had a history of three miscarriages. At birth, APGAR was 8/5/8/10. She was immediately transferred to the neonatal intensive care unit due to respiratory distress and she was intubated and ventilated. On clinical examination, she presented a global hypotonia, without evidence of abnormal movements. Dysmorphic features were noted, including low-set hairline and retrognathia. She had corneal opacities. On the first day of life, laboratory investigations revealed severe hyperlactatemia, with a serum lactate level of 11.9 mM (normal range < 2.2 mM), which persisted between 8 and 9 mM until her death at 4 days. The EEG, performed at H18 of life, showed a disorganized background activity without recorded seizures. Heart ultrasound examination, performed on the third day of life, showed a right ventricular hypertrophy. Brain MRI, performed at two days of life, showed T2 hyperintensity of the white matter, a lactate peak on magnetic resonance spectroscopy (MRS), and bilateral T2 hyperintensity of the thalamus, with no signs of calcifications or hemorrhagic lesions in the basal ganglia. After respiratory deterioration, profound bradycardia, and desaturation, the patient died at 4 days of age. Plasma amino acid chromatography, measured on day 2, showed hyperalaninemia (358 µM, normal range 100-257 µM) and hyperprolinemia (507 µM, normal range 167-345 µM). Urinary amino acid chromatography reveals hyperlactaturia and elevated Krebs cycle intermediates.

Patient 4 was a female born at 35+3 weeks of gestation. Delivery was induced by oxytocin due to abnormal foetal cardiac rhythm. The pregnancy was complicated by IUGR. At birth, the APGAR was 2/5/7/9 and she was intubated and ventilated. At 3 days of life, following a severe hypoglycemic episode (1.2 mM, normal range at 3 days of life >3.3 mM), the patient was transferred to the neonatal intensive care unit. Transthoracic echocardiography revealed dilated cardiomyopathy at day 2. The EEG, performed at day 1, 2 and 3, showed a discontinuous pattern without physiological organisation, and no seizures were recorded. Clinically, she presented clonic movements of the head and the right arm suggestive of focal epileptic seizures. The patient was hypotonic and presented an acute renal failure with anuria since birth, hepatic cytolysis with hepatocellular failure, bilateral congenital cataracts, and persistent hyperlactatemia (during the first check-up, 9 mM, normal range <2.2 mM). A brain MRI could not be performed due to the patient's hemodynamic instability but brain computed tomography (CT), performed at H12, showed hyperdensity of the white matter. The patient deceased at day 3. Amino acid plasmatic chromatography showed elevated alanine levels, without other notable abnormalities. No amino acid urinary chromatography could be performed because of anury. Acylcarnitine profile revealed a moderate increase in propionylcarnitine (0.86 µM, normal range < 0.75 µM).

Patient 5, a boy, was the first child of healthy non-consanguineous parents. During the pregnancy, he presented IUGR. From birth, he exhibited axial hypotonia with hypertonia of the extremities, abnormal movements starting at 8 hours of life, and absence of eye contact. He also had hepatomegaly on ultrasound examination without biological cytolysis, persistent hyperlactatemia and hemolytic anemia. Brain MRI revealed white matter hyperintensity, white matter temporal cysts, and a lactate peak on MR spectroscopy (Figure 1A-B-C-D). Mitochondrial respiratory chain analysis showed deficiencies in complexes I and IV activities in muscle and liver. OXPHOS assembly studied by BN-PAGE on fibroblasts was normal. No mtDNA deletions or mutations were identified. Plasma amino acid chromatography showed a slight elevation of alanine, and urinary chromatography showed elevated fumarate, malate, and lactate. Pyruvate carboxylase activity was normal. He did not undergo an EEG or cardiopulmonary examination. He deceased at 8 days of life.

Patient 6, a girl, was born at 36+3 weeks of gestation to healthy from non-consanguineous parents, and has a healthy sister. The mother underwent a previous pregnancy termination due to severe cardiomyopathy. Her APGAR scores were 10/10. At 1 hour of life, the patient experienced a brief desaturation episode. At 12 hours of life, she presented myoclonic jerks of the lower limbs, along with subsequent hyperlactatemia (at H1, 3.2 mmol/L, H12 14 mmol/L, Day 2, 2.8 mmol/L normal range <2.2 mmol/L). Heart ultrasound examination, performed at 2 days of life, revealed hypertrophic cardiomyopathy. The EEG showed a poor background organisation and bilateral temporo-rolandic spikes (at day 1). During hospitalization, the patient exhibited episodes of clonic legs movement and cyanosis. Brain MRI, performed at day 1, revealed white matter hypersignal in the temporal lobes, on T2 sequences. Plasma amino acid chromatography was normal, while urinary amino acid chromatography showed the presence of methylglutaconic acid. The acylcarnitine profile was normal. The patient death occurs at 14 days of life.

Patient 7. The male foetus of the patient 8's mother presented with bilateral dilated cardiomyopathy. The pregnancy was terminated at 32 weeks SA. A mitochondrial disorder was suspected. Analysis on fibroblasts revealed deficiencies in the assembly of complexes I, II, and IV, as detected by BN-PAGE.

**Supplemental bibliography**

1. Minoche AE, Lundie B, Peters GB, et al. ClinSV: clinical grade structural and copy number variant detection from whole genome sequencing data. *Genome Med*. 2021;13(1):32. doi:10.1186/s13073-021-00841-x

2. Li H, Handsaker B, Wysoker A, et al. The Sequence Alignment/Map format and SAMtools. *Bioinformatics*. 2009;25(16):2078-2079. doi:10.1093/bioinformatics/btp352

3. McKenna A, Hanna M, Banks E, et al. The Genome Analysis Toolkit: A MapReduce framework for analyzing next-generation DNA sequencing data. *Genome Res*. 2010;20(9):1297-1303. doi:10.1101/gr.107524.110

4. Richards S, Aziz N, Bale S, Bick D, Das S, Gastier-Foster J. Standards and guidelines for the interpretation of sequence variants: a joint consensus recommendation of the American College of Medical Genetics and Genomics and the Association for Molecular Pathology. *Genet Med*. Published online May 17, 2015. doi: 10.1038/gim.2015.30.

5. Riggs ER, Andersen EF, Cherry AM, et al. Technical standards for the interpretation and reporting of constitutional copy-number variants: a joint consensus recommendation of the American College of Medical Genetics and Genomics (ACMG) and the Clinical Genome Resource (ClinGen). *Genetics in Medicine*. 2020;22(2):245-257. doi:10.1038/s41436-019-0686-8
